# SARS-CoV-2 Transmission From People Without COVID-19 Symptoms

**DOI:** 10.1001/jamanetworkopen.2020.35057

**Published:** 2021-01-07

**Authors:** Michael A. Johansson, Talia M. Quandelacy, Sarah Kada, Pragati Venkata Prasad, Molly Steele, John T. Brooks, Rachel B. Slayton, Matthew Biggerstaff, Jay C. Butler

**Affiliations:** 1 COVID-19 Response, US Centers for Disease Control and Prevention, Atlanta, Georgia; 2 Office of the Deputy Directory for Infectious Diseases, US Centers for Disease Control and Prevention, Atlanta, Georgia

## Abstract

**Importance:**

Severe acute respiratory syndrome coronavirus 2 (SARS-CoV-2), the etiology of coronavirus disease 2019 (COVID-19), is readily transmitted person to person. Optimal control of COVID-19 depends on directing resources and health messaging to mitigation efforts that are most likely to prevent transmission, but the relative importance of such measures has been disputed.

**Objective:**

To assess the proportion of SARS-CoV-2 transmissions in the community that likely occur from persons without symptoms.

**Design, Setting, and Participants:**

This decision analytical model assessed the relative amount of transmission from presymptomatic, never symptomatic, and symptomatic individuals across a range of scenarios in which the proportion of transmission from people who never develop symptoms (ie, remain asymptomatic) and the infectious period were varied according to published best estimates. For all estimates, data from a meta-analysis was used to set the incubation period at a median of 5 days. The infectious period duration was maintained at 10 days, and peak infectiousness was varied between 3 and 7 days (−2 and +2 days relative to the median incubation period). The overall proportion of SARS-CoV-2 was varied between 0% and 70% to assess a wide range of possible proportions.

**Main Outcomes and Measures:**

Level of transmission of SARS-CoV-2 from presymptomatic, never symptomatic, and symptomatic individuals.

**Results:**

The baseline assumptions for the model were that peak infectiousness occurred at the median of symptom onset and that 30% of individuals with infection never develop symptoms and are 75% as infectious as those who do develop symptoms. Combined, these baseline assumptions imply that persons with infection who never develop symptoms may account for approximately 24% of all transmission. In this base case, 59% of all transmission came from asymptomatic transmission, comprising 35% from presymptomatic individuals and 24% from individuals who never develop symptoms. Under a broad range of values for each of these assumptions, at least 50% of new SARS-CoV-2 infections was estimated to have originated from exposure to individuals with infection but without symptoms.

**Conclusions and Relevance:**

In this decision analytical model of multiple scenarios of proportions of asymptomatic individuals with COVID-19 and infectious periods, transmission from asymptomatic individuals was estimated to account for more than half of all transmissions. In addition to identification and isolation of persons with symptomatic COVID-19, effective control of spread will require reducing the risk of transmission from people with infection who do not have symptoms. These findings suggest that measures such as wearing masks, hand hygiene, social distancing, and strategic testing of people who are not ill will be foundational to slowing the spread of COVID-19 until safe and effective vaccines are available and widely used.

## Key Points

**Question:**

What proportion of coronavirus disease 2019 (COVID-19) spread is associated with transmission of severe acute respiratory syndrome coronavirus 2 (SARS-CoV-2) from persons with no symptoms?

**Findings:**

In this decision analytical model assessing multiple scenarios for the infectious period and the proportion of transmission from individuals who never have COVID-19 symptoms, transmission from asymptomatic individuals was estimated to account for more than half of all transmission.

**Meaning:**

The findings of this study suggest that the identification and isolation of persons with symptomatic COVID-19 alone will not control the ongoing spread of SARS-CoV-2.

## Teaser

This decision analytical model assesses the proportion of severe acute respiratory syndrome coronavirus 2 (SARS-CoV-2) transmissions in the community that likely occur from persons without symptoms.

## Introduction

As severe acute respiratory syndrome coronavirus 2 (SARS-CoV-2), the novel coronavirus that causes coronavirus disease 2019 (COVID-19), began to spread globally, it became apparent that the virus, unlike the closely related SARS-CoV in the 2003 outbreak, could not be contained by symptom-based screening alone. Asymptomatic and clinically mild infections were uncommon during the 2003 SARS-CoV outbreak, and there were no reported instances of transmission from persons before the onset of symptoms.^1^ SARS-CoV-2 spread faster than SARS-CoV, and accumulating evidence showed that SARS-CoV-2, unlike SARS-CoV, is transmitted from persons without symptoms. However, measures to reduce transmission from individuals who do not have COVID-19 symptoms have become controversial and politicized and have likely had negative effects on the economy and many societal activities. Optimal control of COVID-19 depends on directing resources and health messaging to mitigation efforts that are most likely to prevent transmission. The relative importance of mitigation measures that prevent transmission from persons without symptoms has been disputed. Determining the proportion of SARS-CoV-2 transmission that occurs from persons without symptoms is foundational to prioritizing control practices and policies.

Transmission by persons who are infected but do not have any symptoms can arise from 2 different infection states: presymptomatic individuals (who are infectious before developing symptoms) and individuals who never experience symptoms (asymptomatic infections, which we will refer to as never symptomatic). Early modeling studies of COVID-19 case data found that the generation interval of SARS-CoV-2 was shorter than the serial interval, indicating that the average time between 1 person being infected and that person infecting someone else was shorter than the average time between 1 person developing symptoms and the person they infected developing symptoms.^2,3,4,5^ This finding meant that the epidemic was growing faster than would be expected if transmission were limited to the period of illness during which individuals were symptomatic. By the time a second generation of individuals was developing symptoms, a third generation was already being infected. Epidemiological data from early in the pandemic also suggested the possibility of presymptomatic transmission,^6,7^ and laboratory studies confirmed that levels of viral RNA in respiratory secretions were already high at the time of symptom onset.^8,9,10^

Asymptomatic SARS-CoV-2 transmission also occurs because of individuals with infection who are never symptomatic (or who experience very mild or almost unrecognizable symptoms). The proportion of individuals with infection who never have apparent symptoms is difficult to quantify because it requires intensive prospective clinical sampling and symptom screening from a representative sample of individuals with and without infection. Nonetheless, evidence from household contact studies indicates that asymptomatic or very mild symptomatic infections occur,^11,12,13,14^ and laboratory and epidemiological evidence suggests that individuals who never develop symptoms may be as likely as individuals with symptoms to transmit SARS-CoV-2 to others.^9,15,16^

## Methods

The Centers for Disease Control and Prevention determined that this decision analytical study, which involved no enrollment of human subjects, did not require institutional review board approval. We used a simple model to assess the proportion of transmission from presymptomatic (ie, infectious before symptom onset), never symptomatic, and symptomatic individuals across a range of scenarios in which we varied the timing of the infectious period to assess different contributions of presymptomatic transmission and the proportion of transmission from individuals who never develop symptoms (ie, remain asymptomatic).

For all estimates we used data from a meta-analysis of 8 studies from China to set the incubation period at a median of 5 days with 95% of symptomatic individuals developing symptoms by day 12.^17^ Therefore the daily (*t*) probability of symptom onset (*p_so_*) for individuals who develop symptoms was:

*p_so_*(*t*) = *F_Log−Normal_*(*t*,logmean = 1.63,logsd = 0.5).

To approximate a distribution of the infectious period, we made a baseline assumption that peak infectiousness occurs on average at the same time as the median incubation period, such that infectiousness begins prior to symptom onset (Table).^9,12,14,15,16,18,20^ We then assumed that infectiousness (*I*) over time can be approximated by a γ density function and that the average person is infectious for as long as approximately 10 days (ie, 98% of transmission happens within a 10-day period)^11^:

*I(t)* = *f_γ_*(*t*,mode = 5,interval = 10).

For all estimates, we maintained the infectious period duration as 10 days, but varied the mode between 3 and 7 days (−2 and +2 days relative to the median incubation period).

**Table.** Key Assumptions and Evidence Informing Those Assumptions

Source	Evidence base	Estimate or assumption
**Assumptions for presymptomatic transmission**		
Peak infectiousness relative to onset, d		
Casey et al, 2020^18^	Range, 17 studies	−3 to 1.2 d
Assumed baseline	NA	0 d
Assumed range	NA	−2 to 2 d
**Assumptions for never symptomatic transmission**		
Proportion never symptomatic		
Oran et al, 2020^12^	Inferred range, 16 studies	30% to 45%
Buitrago-Garcia et al, 2020^14^	Meta-estimate, 7 studies	26% to 37%
Davies et al, 2020^20^	Age-dependent estimate, 6 studies	20% to 70%
Assumed baseline	NA	30%
Relative infectiousness of individuals who never have symptoms		
Lee et al, 2020^9^	303 patients, assessment of viral shedding	Approximately 100%
Chaw et al, 2020^15^	1701 secondary contacts	40% to 140%
Mc Evoy et al, 2020^16^	Inferred range, 6 studies	40% to 70%
Assumed baseline		75%
Overall proportion of individuals who never have symptoms transmission		
Assumed range	NA	0% to 70%

Abbreviation: NA, not applicable.

Uncertainty also remains about the proportion of individuals with infection who are never symptomatic (*p_ns_*) and the relative contribution of these infections to transmission (*r_ns_*). Estimates of *p_ns_* range from single digits to more than 50%, many with potential biases related to the study population (eg, age, prevalence of comorbidities) and the extent of long-term follow-up^12,13,14,19,20^ (Table). We made a baseline assumption that 30% of individuals with infection are never symptomatic and then assessed higher or lower assumptions. We also made a baseline assumption that individuals with asymptomatic infections are on average 75% as infectious as those with symptomatic infections.^9,15,16^ Combined, these baseline assumptions imply that persons with infection who never develop symptoms may account for approximately 24% of all transmission (*T*):

*T_ns_* = *p_ns_* × *r_ns_* / (*p_ns_* × *r_ns_* + [1 − *p_ns_*]).

We varied this overall proportion, T*_ns_*, between 0% and 70% to assess a wide range of possible proportions. The daily proportion of transmission from individuals after symptom onset (*T_s_*) was therefore:

*T_s_*(*t*) = (1 − *T_ns_*) × *p_so_*(*t*) × *I*(*t*),

and the daily proportion of transmission from presymptomatic (*T_ps_*) individuals, ie, those who develop symptoms but become infectious prior to symptom onset, is:

*T_ps_*(*t*) = 1 − *T_s_*(*t*) − T*_ns_*.

We modified baseline assumptions to consider the relative importance of different levels of never symptomatic and presymptomatic transmission. Code is available in the eAppendix in the Supplement.

### Statistical Analysis

All analyses were conducted in R version 4.0.1 (R Project for Statistical Computing). No statistical testing was conducted, so no prespecified level of significance was set.

## Results

**Figure 1.**
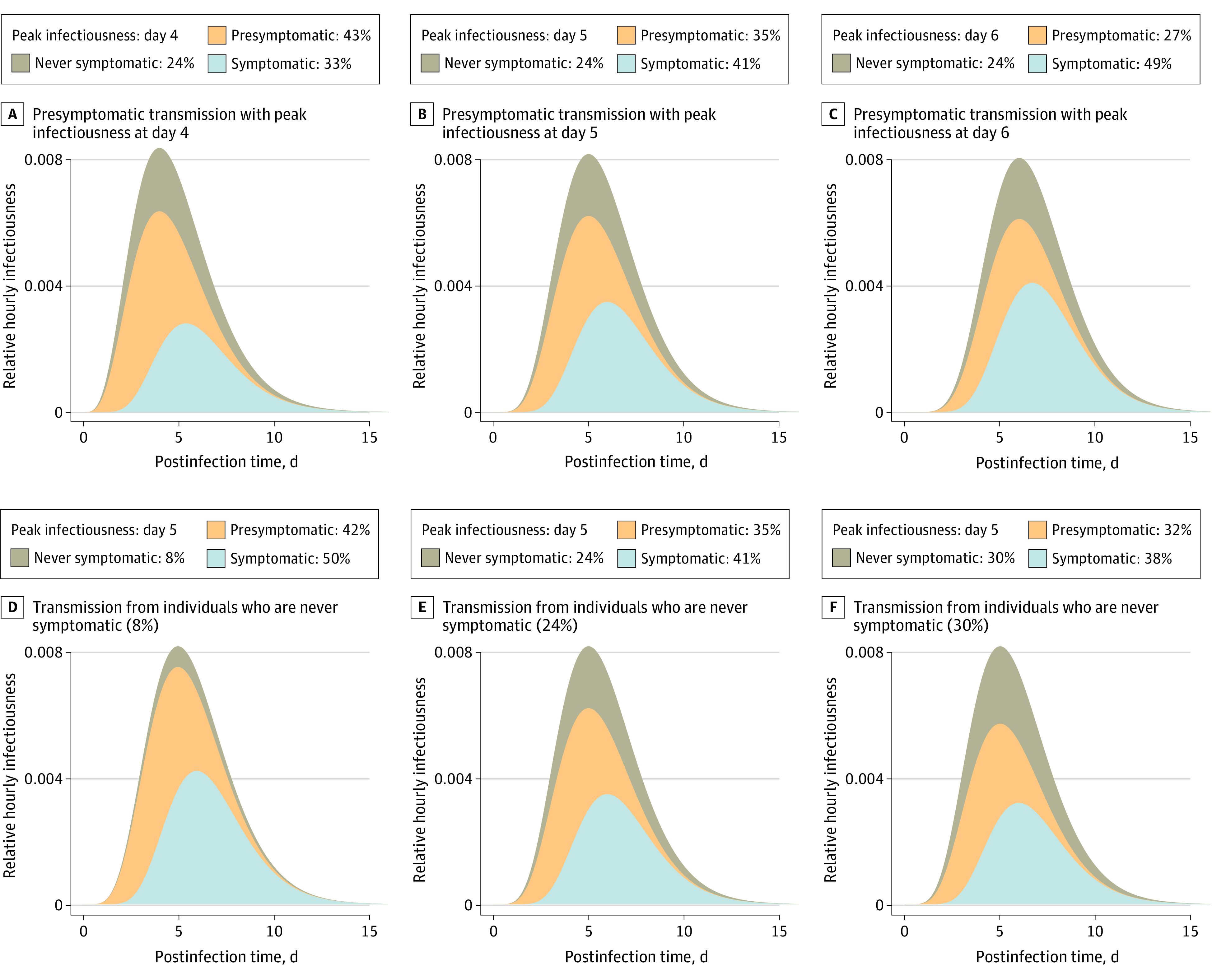
The Contribution of Asymptomatic Transmission Under Different Infection Profiles The top curve in each panel represents the average relative hourly infectiousness, such that while the lower curves change under different assumptions, the total hourly infectiousness equals 1 in all cases. Within each curve, the colored area indicates the proportion of transmission from each class of individuals. The portion attributed to individuals with symptoms (light blue) can also be interpreted as the maximum proportion of transmission that can be controlled by immediate isolation of all symptomatic cases. Panels A, B, and C show different levels of presymptomatic transmission. We calibrated infectiousness to peak at day 4 (A), 5 (B; median incubation period), or 6 (C) days. Panels D, E, and F show different proportions of transmission from individuals who are never symptomatic: 8% (C; eg, 10% never symptomatic and 75% relative infectivity), 24% (D; baseline, 30% never symptomatic and 75% relative infectivity), and 30% (E; eg, 30% never symptomatic and 100% relative infectivity).

Under baseline assumptions, approximately 59% of all transmission came from asymptomatic transmission: 35% from presymptomatic individuals and 24% from individuals who are never symptomatic (Figure 1). Because each component is uncertain, we assessed different timings of peak infectiousness relative to illness onset and different proportions of transmission from individuals who never have symptoms. Maintaining the 24% of transmission from individuals who never have symptoms, but shifting peak infectiousness 1 day earlier (to day 4) increased presymptomatic transmission to 43% and all asymptomatic transmission to 67% (Figure 1A). A later peak (ie, day 6) decreased presymptomatic to 27% and all asymptomatic transmission to 51% (Figure 1C).

Holding the day of peak infectiousness constant at day 5 and decreasing the proportion of transmission from individuals who are never symptomatic to 10% with a relative infectiousness of 75% (baseline assumption), the proportion of all transmission from those who are never symptomatic decreased to 8%, presymptomatic transmission increased to 42%, and combined asymptomatic transmission was 50% of all transmission (Figure 1D). In contrast, if the proportion of those who ever develop symptoms was 30% and their relative infectiousness increased to 100%, they contributed 30% of all transmission, presymptomatic transmission was 32%, and combined asymptomatic transmission was 62% of all transmission (Figure 1F).

**Figure 2.**
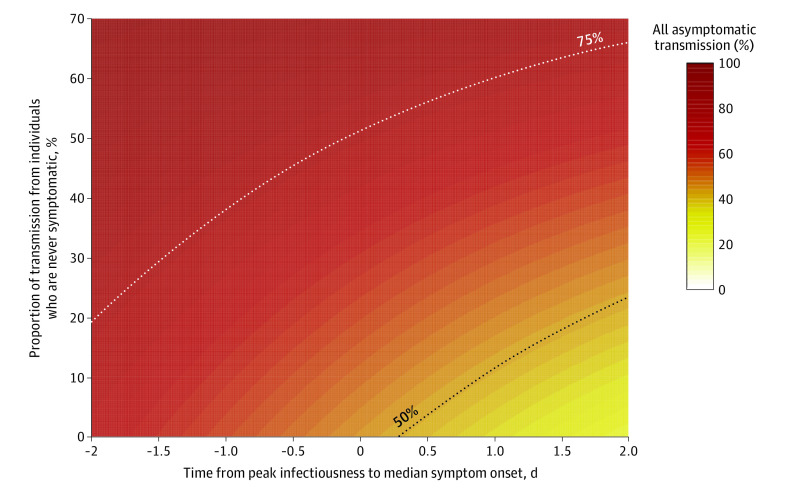
Combined Transmission From Individuals Who Are Presymptomatic and Those Who Never Have Symptoms Colors indicate the proportion of transmission due to all individuals without symptoms at the time of transmission, including presymptomatic transmission (x-axis, the timing of peak infectiousness relative to symptom onset) and transmission from individuals who are never symptomatic (y-axis). For example, peak infectiousness at the same time as median symptom onset (0 days difference) with 10% of transmission from individuals who never have symptoms would mean that approximately 51% of transmission is from asymptomatic individuals.

Uncertainty remains regarding the magnitude of both presymptomatic and never symptomatic transmission. Therefore, we analyzed a wider range of each of these components, with peak infectiousness varying between 2 days before (more presymptomatic transmission) to 2 days after (less presymptomatic transmission) median symptom onset and with never symptomatic transmission ranging from 0% to 70% (Figure 2). Under this broader range of scenarios, most combined assumptions of peak infectiousness timing and transmission from individuals who never have symptoms indicated that at least 50% of new SARS-CoV-2 infections likely originated from individuals without symptoms at the time of transmission. If more than 30% of transmission was from individuals who never have symptoms, total asymptomatic transmission was higher than 50% with any value of peak infectiousness, up to 2 days after the median time of symptom onset. If peak infectiousness was at any point approximately 6 hours before median symptom onset time, more than 50% of transmission was from individuals without symptoms, regardless of the proportion from those who never have symptoms. Even a very conservative assumption of peak infectiousness 2 days post–median onset and 0% never symptomatic transmission still resulted in more than 25% of transmission from asymptomatic individuals.

## Discussion

The findings presented here complement an earlier assessment^21^ and reinforce the importance of asymptomatic transmission: across a range of plausible scenarios, at least 50% of transmission was estimated to have occurred from persons without symptoms. This overall proportion of transmission from presymptomatic and never symptomatic individuals is key to identifying mitigation measures that may be able to control SARS-CoV-2. For example, if the reproduction number (*R*) in a given setting is 2.0, then at least a 50% reduction in transmission is needed to drive the reproductive number below 1.0. Given that in some settings *R* is likely much greater than 2 and more than half of transmissions may come from individuals who are asymptomatic at the time of transmission, effective control must mitigate transmission risk from people without symptoms.

### Limitations

This study has limitations. First, we used a simplistic model to represent a complex phenomenon, ie, the average infectiousness of SARS-CoV-2 infections over time. We used this model deliberately to test assumptions about the timing of peak infectiousness and transmission among asymptomatic individuals so that we could vary only these 2 critical parameters and assess their relative effects. Therefore, these results lack quantitative precision, but they demonstrate the qualitative roles of these 2 components and show that across broad ranges of possible assumptions, the finding that asymptomatic transmission is a critical component of SARS-CoV-2 transmission dynamics remains constant.

As discussed here, the exact proportions of presymptomatic and never symptomatic transmission are not known. This also applies to the incubation period estimates, which are based on individual exposure and onset windows that are difficult to observe with precision and therefore include substantial uncertainty even when leveraging estimates across multiple studies. Moreover, they likely vary substantially in different populations. For example, older individuals are more likely than younger persons to experience symptoms,^20^ so in populations of older individuals, never asymptomatic transmission may be less important. However, specific age groups are rarely exclusively isolated from other age groups, so asymptomatic transmission risk is still important in those groups and even more so in younger age groups, in which transmission may be even more dominated by asymptomatic transmission.^20^

Real-world transmission dynamics are also not entirely dependent on the individual-level dynamics of infectiousness over time. Now that COVID-19 is widely recognized, individuals with COVID-19 symptoms are more likely to isolate themselves and further reduce the proportion of transmission from symptomatic individuals, shifting a greater proportion of transmission to those who do not have symptoms. In this sense, the estimates here represent the lower end of the proportion of asymptomatic transmission in the presence of interventions to reduce symptomatic transmission.

## Conclusions

Under a range of assumptions of presymptomatic transmission and transmission from individuals with infection who never develop symptoms, the model presented here estimated that more than half of transmission comes from asymptomatic individuals. In the absence of effective and widespread use of therapeutics or vaccines that can shorten or eliminate infectivity, successful control of SARS-CoV-2 cannot rely solely on identifying and isolating symptomatic cases; even if implemented effectively, this strategy would be insufficient. These findings suggest that effective control also requires reducing the risk of transmission from people with infection who do not have symptoms. Measures such as mask wearing and social distancing empower individuals to protect themselves and, if infected, to reduce risk to their communities.^21^ These measures can also be supplemented by strategic testing of people who are not ill, such as those who have exposures to known cases (eg, contact tracing) or are at high risk of exposing others (eg, congregate facility staff, those with frequent contact with the public). Multiple measures that effectively address transmission risk in the absence of symptoms are imperative to control SARS-CoV-2.

## Notes

Supplement. **eAppendix.** Code for Analysis
