# Error in Supplement

**DOI:** 10.1001/jamanetworkopen.2021.1383

**Published:** 2021-02-12

In the Original Investigation titled “SARS-CoV-2 Transmission From People Without COVID-19 Symptoms,”^1^ published January 7, 2021, there was an error in the Supplement. The code in the eAppendix was incorrect, and it has been replaced.^1^
