# Supplementary material for: SARS-CoV-2 Transmission From People Without COVID-19 Symptoms
Source: JAMA Netw Open. 2021 Jan 7;4(1):e2035057. doi: 10.1001/jamanetworkopen.2020.35057 (PMC7791354; doi:10.1001/jamanetworkopen.2020.35057)
Supplement: Supplement. — eAppendix. Code for Analysis [file jamanetwopen-e2035057-s001.pdf]

Supplemental Online Content

Johansson MA, Quandelacy TM, Kada S, et al. SARS-CoV-2 transmission from people without COVID-19 symptoms. *JAMA Netw Open*. 2021;4(1):e2035057. doi:10.1001/jamanetworkopen.2020.35057

eAppendix 1. Code for Analysis

This supplemental material has been provided by the authors to give readers additional information about their work.

© 2021 Johansson MA et al. *JAMA Network Open.*

eAppendix. Code for Analysis

The analyses were run in R (r-project.org) using the following code.

```
# incubation period
p_symp_onset <- function(t, logmu_inc=1.63, logsd_inc=0.5) {
  plnorm(t, logmu_inc, logsd_inc)
}

# relative infectiousness over time
infectiousness_daily <- function(t, t_peak_inf, days_inf) {
  optim_duration <- function(scale) {
    shape <- t_peak_inf / scale + 1
    abs(diff(qgamma(c(0.01, 0.99), shape=shape, scale=scale)) - days_inf)
  }
  scale <- optimize(optim_duration, c(0.1, 10))$minimum
  shape <- t_peak_inf / scale + 1
  dgamma(t, shape=shape, scale=scale)
}

# proportion of transmission from never symptomatic
trans_never_symp <- function(prop_never_symp, rel_inf_never_symp) {
  (prop_never_symp * rel_inf_never_symp) /
    (prop_never_symp * rel_inf_never_symp + (1 - prop_never_symp))
}

### Example calculations
times <- 0:100
trans_never_symp_baseline <-
  trans_never_symp(prop_never_symp=0.3, rel_inf_never_symp=0.75)
t_peak_inf <- 5
days_inf <- 10

prop_symptomatic <- (1 - trans_never_symp_baseline) *
  sum(p_inc(times) * infectious_g(times, t_peak_inf=t_peak_inf, days_inf=days_inf))

# Never symptomatic
prop_never_symptomatic

# Symptomatic
prop_symptomatic

# Pre-symptomatic
1 - prop_never_symptomatic - prop_symptomatic
```

© 2021 Johansson MA et al. *JAMA Network Open.*
